# Recurrent Catatonia due to Episodic Obsessive-Compulsive Disorder

**DOI:** 10.1155/2022/2022474

**Published:** 2022-11-15

**Authors:** Soumitra Das, Sakshi Prasad, Palak Atul Fichadia, Abhigan Babu Shrestha, Ozge C. Amuk Williams, Anil Bachu

**Affiliations:** ^1^Emergency Mental Health, Sunshine Hospital, Melbourne, Australia; ^2^Faculty of Medicine, National Pirogov Memorial Medical University, 21018 Vinnytsya, Ukraine; ^3^Smt. NHL Municipal Medical College, 380006 Gujarat, India; ^4^M Abdur Rahim Medical College, Dinajpur, Bangladesh; ^5^Griffin Memorial Hospital, Norman, Oklahoma, USA; ^6^Department of Psychiatry, Baptist Health-UAMS Combined Program, Little Rock, Arkansas, USA

## Abstract

**Background:**

Catatonia is regarded as a diverse type of motor dysregulation syndrome that includes mutism, immobility, catalepsy, negativism, stereotypies, and echo phenomena. Catatonia is known to coexist with a wide range of physical and mental health conditions, including mood disorders, schizophrenia, autoimmune disorders, and metabolic abnormalities. Albeit, the association between obsessive-compulsive disorder (OCD) and catatonia is underreported, and mechanisms are not well elucidated. *Study*. In this study, we present a case of a 36-year-old woman who developed episodes of catatonia during the course of her obsessive-compulsive disorder (OCD). Success rates have been recorded with both benzodiazepines and electroconvulsive therapy (ECT). Gauging the severity of her symptoms and poor drug compliance, the patient was opted for and successfully treated with ECT. She was also educated about OCD through a series of therapy sessions and exposure and response prevention (ERP) principles. She was maintained on ERP and adjunctive clonazepam upon discharge. On subsequent follow-ups, the patient seemed to be doing well and was eager to begin her job again.

**Conclusion:**

Our study shows a possible link between OCD and catatonia. Additionally, robust studies are needed in order to determine the pathophysiology of catatonia and the mechanism of ECT so that more beneficial therapeutics can be developed. A combination of ECT and antidepressants with ERP therapy for recurrent catatonia with OCD could be effective as a therapeutic modality.

## 1. Introduction

Catatonia is regarded as a diverse type of motor dysregulation syndrome that includes mutism, immobility, catalepsy, negativism, stereotypies, and echo phenomena [1]. More than 10% of patients with acute psychiatric conditions have been found to experience this psychomotor condition [1]. The syndrome has been divided into two subtypes. Retarded-type catatonia is marked by rigidity, immobility, staring, mutism, and a variety of other clinical symptoms. In a less frequent condition known as excited catatonia, patients experience protracted episodes of psychomotor agitation. Catatonia, once believed to be a subset of schizophrenia, is now known to coexist with a wide range of physical and mental health conditions, including affective disorders like depression, bipolar disorder, and schizophrenia and medical conditions like encephalitis, autoimmune disorders, strokes, intracranial mass lesions, vitamin B12 deficiency, and Wilson disease, and as a consequence of other drugs like psychotropic drugs, including fluphenazine, haloperidol, risperidone, and clozapine, and nonpsychotropic drugs such as steroids, disulfiram, ciprofloxacin, and several benzodiazepines [2].

In many cases, catatonia must be treated before a precise diagnosis of any underlying issues can be made [3]. There are however many unanswered questions regarding the connection between OCD and catatonia, which makes it difficult to diagnose and treat patients who suffer from both diseases [4].

The fact that catatonic syndrome is linked to other illnesses highlights the urgency of a prompt diagnosis and course of action. For instance, the development of neuroleptic malignant syndrome, which has a mortality rate of about 10% and may be clinically indistinguishable from malignant catatonia, appears to be a risk factor for catatonia. Catatonia itself can make it difficult, if not impossible, to conduct patient interviews and physical tests, making it harder to identify underlying diseases. These side effects of catatonia emphasize how critical it is to identify the condition and start treatment as soon as possible [1].

The cornerstone of curing disease is proper diagnosis. Unlike medical or surgical diseases, mental disorders are substantially symptom-based diagnoses. According to the Diagnostic and Statistical Manual of Mental Disorders (DSM-5) or the International Classification of Diseases, Tenth Revision, Clinical Modification (ICD-10), in the process of evaluating, syndromes are invariably associated with certain diagnoses. Hence, although rare, catatonia may be associated with obsessive-compulsive disorder (OCD) [5, 6].

Benzodiazepines are considered first-line treatments for catatonia. However, only 70% and 79% of cases remit with benzodiazepines like lorazepam [7]. In refractory cases with medical therapy, the use and efficacy of electroconvulsive therapy (ECT) are bolstered by limited case studies [8, 9]. In this paper, we present a case of a 36-year-old woman who developed episodes of catatonia during the course of her obsessive-compulsive disorder (OCD). Success rates have been recorded with both benzodiazepines and electroconvulsive therapy (ECT). Gauging the severity of her symptoms and poor drug compliance, the patient was opted for and successfully treated with ECT. This report has been drafted in accordance with CARE guidelines [10].

## 2. Case Presentation

A 36-year-old female was brought to a psychiatric inpatient unit by her children (male 12 years old and female 14 years old) with complaints of slowness in activities, withdrawn behavior, slow speaking, and blank staring for the past 7-8 weeks. She has a history of separation from her husband and is unemployed for 2 years. Her past psychiatric history comprised of a 13-year history of OCD and unremarkable history of alcohol and drug abuse. Her children's report suggested medication noncompliance and an insignificant family history for behavioral health issues. Her activities of daily living including the ability to work, look after her children, and caring for herself were completely compromised. She scored 24 with the Bush-Francis Catatonia Rating Scale (BFCRS), with immobility, mutism, staring, posturing, grimacing, negativism, withdrawal, and ambitendency. Citing the severity of her condition and inability to consume food, she was started on ECT with a threshold of 60 mC (millicoulombs) and an average seizure duration of 25-50 secs. After 3 ECT sessions, her BFCRS score dropped to 4 within a span of 1 week. She then only had negativism, mutism, and immobility. Her condition improved further during the coming days.

She, however, reported that she was not talking as she talks before the episode of catatonia, had recurrent negative thoughts, and had a strong urge to utter obscene words. She was afraid to open her mouth and speak at all. Her behavior was observed to be of obsessive nature. She spent several hours obsessing over her thoughts, moderate distress, impairment, and control of her obsession scoring 12 on the obsession scale. Out of compulsion for the same, she decided to stop speaking altogether, avoided social gatherings out of fear, dragged her leg, and tapped her fingers in view of controlling it.

Following a complete recovery from her catatonic state, she expressed extreme regret, depressed mood, guilt, and worthlessness. She was eventually treated for a brief episode of depression. She confided that her urge of blurting obscene language and recurrent negative thoughts began every time before the episode of catatonia and had experienced 3 such episodes in the past 13 years, with each episode of depression following her OCD. She improved with each ECT session and was subsequently mantained on SSRI. She stopped the medications after a few months following improvement. Her last episode was 10-11 months prior to the latest episode. After more elaborate and repeated case histories, it was found that her previous depressive symptoms used to be for 6-8 months with OCD symptoms of 1-4 weeks. This time, it was reversed with depression of 2 weeks and OCD of 7-8 weeks. Considering her poor drug compliance, distress and self-guilt, and lack of knowledge about her condition, she was also started on ERP (exposure and response prevention). The first few days proved to be the most challenging for both the patient and the clinical psychologist, as her compulsive thoughts forced her to stop speaking out of fear with the psychologist. With adjunctive clonazepam, she was educated about OCD and ERP principles and maintained on ERP. On subsequent follow-ups, the patient seemed to be doing well and was eager to begin working again.

## 3. Discussion

To our knowledge, only fourteen case reports have been published concerning catatonia with OCD manifestation [4, 8, 9, 11–20], among which only four articles have shown ECT efficacy for recurrent catatonia [9, 13, 15, 17]. A study by D'Urso et al. showed successful treatment of catatonia and OCD, whereas Duarte-Batista et al. in their study depicted transient improvement of catatonia, eventually requiring deep brain stimulation [8, 9]. In our study, we present effective management of recurrent catatonia using ECT. However, despite the use of antidepressants and ERP therapy, OCD was not successfully treated. In a meta-analysis conducted by Pluijms et al., the efficacy of ECT for major depression improved significantly with an adjuvant antidepressant [21]. Additionally, our patient displayed depressive symptoms; she described signs and symptoms consistent with a major depressive disorder diagnosis after ECT, and an SSRI helped her recover from catatonia. It was noted that in the past 13 years, there have been three instances of recovery from catatonia followed by closely spaced episodes of depression and OCD. In her first two episodes, depression persisted for 6–8 months while OCD persisted for 1-4 weeks. However, this pattern of depression followed by OCD appeared to be reversed in her most recent episode of catatonia, where the depression persisted for 2 weeks and OCD for 7 weeks.

There are many parameters for measuring catatonia, of which the most commonly used is the Bush-Francis Catatonia Rating Scale. After receiving treatment, our patient's score decreased from 24 at the time of presentation, which indicated severe catatonia, to 4, indicating a marked improvement in her symptoms. She now only exhibited negativism, mutism, and immobility [22].

Pharmacological management used for catatonia entails targeting *γ*-amino-butyric acid- (GABA-) A, glutamate, and dopamine, thus hinting at the possibility of dysfunction in these neurotransmitter systems as the causal factor in catatonia [23, 24].

Depletion of cortical GABA had been noticed in catatonia and is hypothesized to change basal ganglia modulation and provoke motor symptoms [25]. This could explain the dramatic therapeutic effect of benzodiazepines, which quickly reverse catatonic symptoms because of the normalization of regulatory circuits [26, 27]. Serotonin exerts an inhibitory effect over dopamine in all brain areas [28]. Also, dopaminergic hyperactivity is anticipated to occur in conditions correlated with serotonergic system hypofunction, like major depression, PTSD (posttraumatic stress disorder), panic disorder, and social anxiety disorder. Another condition that is associated with serotonergic hypofunction is OCD [29]. Based on all this evidence, contingency of catatonia in OCD seems workable.

Benzodiazepines are considered the first-line therapy for catatonia and ECT as the second line. The exact mechanism of ECT has not been discovered, and there is a paucity of literature on the role of ECT in catatonia. However, there is no doubt about the therapeutic efficacy of ECT in catatonia [30].

When a benzodiazepine (BZP) does not work as well as it should or there is a serious risk of severe morbidity or mortality, ECT may be used to treat catatonia. With BZPs, catatonia can respond favorably, as is widely documented. Due to their accessibility and convenience of usage, this class of agents is frequently used as a first-line intervention. Nevertheless, only about 70% of catatonia cases react to BZPs. Therefore, ECT may also be taken into account when catatonia is detected [30].

A patient receiving a BZP may also be receiving simultaneous process of getting ready for ECT. ECT must be seriously considered if, after five days of high-dose BZP therapy, there has been little to no improvement, no improvement at all, or if there are signs of fatal catatonia developing (e.g., fever, changes in blood pressure and heart rate, and rising levels of creatinine phosphokinase). The continuation of BZP use is not prohibited once ECT is started. It has been established that lorazepam and ECT are effective for treating catatonia. When given before the induction of anesthesia for ECT, a BZP receptor antagonist like flumazenil can fast reverse BZPs [31]. To conclude the findings in Table 1, first-line choice is a medical approach with anti-OCD therapy comprising mostly of neuroleptics. In addition, combination treatment of psychotherapy and neuroleptics has proven to be the most effective treatment regimen. If still not controlled, then ECT may be considered. Throughout each step, behavioral and/or psychotherapy should be added for better outcome.

As catatonia is linked with other mental disorders, it makes it difficult to diagnose it accurately and in a timely manner. Studies have also shown ample cases of undiagnosed catatonia [32, 33]. Recognizing and treating catatonia usually result in rapid resolution of the syndrome, whereas failing to recognize it may lead to potentially fatal complications including infection, neuroleptic malignant syndrome, pulmonary embolism, and dangerous medical complications like pressure sores, nutritional and electrolyte disturbances, venous thrombosis, muscle contractures, and aspiration pneumonia [1, 34]. Before effective treatment strategies were developed, mortality rates approached 50% in cases of “lethal catatonia” as a result of medical complications associated with the syndrome. The current response rate of acute catatonia to first-line treatments (i.e., benzodiazepines and ECT) varies from 70% to 85%, and cases of treatment-refractory chronic catatonia are rare [3].

Catatonia itself can make it difficult, if not impossible, to conduct patient interviews and physical tests, making it harder to identify underlying diseases. These side effects of catatonia emphasize how critical it is to identify the condition and start treatment as soon as possible [1, 3].

## 4. Conclusion

Our study shows a possible link between OCD and catatonia. Additionally, robust studies are needed in order to determine the pathophysiology of catatonia and the mechanism of ECT so that more beneficial therapeutics can be developed. A combination of ECT and antidepressants with ERP therapy for recurrent catatonia with OCD could be effective as a therapeutic modality. Besides, as a subset of OCD patients' fixation compounds, they become more susceptible to catatonia [4]. Therefore, those who are catatonic should be evaluated for underlying OCD.

## Figures and Tables

**Table 1:** The treatment interventions and outcomes of individual cases.

Case report	Country and year of study	Treatment intervention	Outcome
D'Urso et al. [9]	Italy, 2012	ECT with clonazepam, paroxetine, and perphenazine	(i) BPRS (Brief Psychiatric Rating Scale) score decreased by 49% (from 79 to 40); CGI-severity item changed from “among the most extremely ill (7/7)” to “markedly ill (5/7)” (ii) The core component of the same scale showed (iii) A 42% reduction of obsessive-compulsive symptoms (from 38 to 22) (iv) Hamilton Depression Rating Scale (HAM-D) score decreased from 21 to 9 (57%), and Hamilton Anxiety Rating Scale decreased (HAM-A) from 19 to 10 (47%)

Duarte-Batista et al. [8]	Portugal, 2020	Bilateral DBS of the anterior limb of the internal capsule (ALIC)/bed nucleus of stria terminalis (BST) region was performed, using a target below the BST and a trajectory through the ALIC, with stimulation of contacts 0 and 3	(i) Two weeks after surgery, sedatives were suspended, and the patient was successfully extubated (ii) One year after surgery, the patient reached a YGTSS (Yale Global Tic Severity Scale) of 19, representing an 81% improvement. OCD is completely resolved (iii) Adverse events were a superficial infection and weight gain (iv) In conclusion, this ALIC/BST stimulation appears to have been an effective and safe treatment for Gilles de la Tourette syndrome (GTS) with OCD in this case

Hermesh et al. [14]	Israel, 1989	In one instance, clomipramine was utilized, and in another, behavior therapy	(i) Neuroleptics were ineffective in treating catatonic symptoms, whereas traditional OCD treatments were effective

Fontenelle et al. [4]	Brazil, 2007	Antiobsessional drugs and anticatatonia measures	(i) Treatment plan for patients with OCD and comorbid catatonia entails a number of steps, like fine-tuning the antiobsessional therapy, managing cooccurring disorders that may lead to catatonia, stopping, and then slowly restarting medications

Jaimes-Albornoz et al. [16]	Spain, 2021	OCD treatment	(i) Optimization of OCD treatment helped to resolve symptoms of catatonia

Mukai et al. [18]	USA, 2011	Aripiprazole, memantine, and lorazepam were among the psychopharmacological medications used. Addition of fluvoxamine to target obsessive-compulsive disorder- (OCD-) like symptoms A thorough medical examination identified a cervical spine hemangioma, which was surgically removed and improved neck posture	Clinical improvement was seen after adding fluvoxamine to treat obsessive-compulsive disorder- (OCD-) like symptoms, pointing to OCD as a potential contributor to this patient's protracted catatonic condition

Nikjoo et al. [19]	USA, 2022	Lorazepam	Catatonic symptoms were successfully treated at the expense of developing a subtype of OCD known as scrupulosity

Blacker [11]	USA; 1966	Psychotherapy, phenothiazine	Improvement over the course of 5 years

Eryilmaz et al. [13]	Turkey, 2014	Aripiprazole, clozapine, fluvoxamine, clonazepam, and ECT therapy were used	(i) Pharmacotherapy was carried out as aripiprazole 30 mg per day, biperiden 4 mg per day, and pimozide 2 mg per day. ECT was begun because of no responsiveness to pharmacotherapy (ii) After the third session of ECT, recurrent ritual behavior and posturing were observed (iii) The patient had obsessions such as trying not to forget thoughts in case they become needed and being able to pass to another thought after touching things. Pimozide was discontinued (iv) Aripiprazole dose was decreased to 20 mg per day. Fluvoxamine 100 mg per day and clonazepam 6 mg per day were added to the treatment regime. ECT was discontinued after the 10th session (v) The patient was discharged with partial remission on aripiprazole 20 mg per day, clonazepam 2 mg per day, and fluvoxamine 200 mg per day

Elia et al. [12]	USA, 2005	(i) Plasmapheresis (ii) Lorazepam	(i) OCD symptoms significantly and quickly improved after plasmapheresis, and basal ganglia edema also decreased, which is consistent with an immune-mediated pathophysiological process involving group A beta-hemolytic streptococci (ii) The symptoms of attention-deficit/hyperactivity disorder may be signs of catatonia as impulsivity, hyperactivity, and inattention decreased with lorazepam

Jagadheesan et al. [15]	India, 2002	Patient 1: for catatonic signs, injection lorazepam For OCD with catatonia, a combination of clomipramine and risperidone subsequently combined clomipramine, thioridazine, and buspirone Patient 2: for catatonic schizophrenia, electroconvulsive therapy (ECT). Then, amitriptyline and lithium, with the second trial of ECT and a combination of imipramine and trifluoperazine	Patient 1: after lorazepam, symptoms were not relieved, and depression was noted. Then, with initial combination therapy, the symptoms worsened. Subsequent combination therapy relieved the symptoms Patient 2: initial ECT and combination therapy were inadequate to treatment. With the addition of a further second trial of ECT, drugs responded well

Sachdeva et al. [20]	India, 2015	Trifluoperazine, fluoxetine, trihexyphenidyl, and phenytoin	(i) With combination therapy, the patient showed significant improvement over the subsequent six weeks of admission; the Brief Psychiatric Rating Scale (BPRS) dropped from 42 to 24 (ii) The Yates-Brown Obsessive Compulsive Symptoms (YBOCS) scale dropped from 24 to 18 (iii) The Global Assessment of Functioning (GAF) scale increased from 25 to 55 (iv) After 6 months of discharge, the patient had good improvement

Makhinson et al. [17]	USA, 2012	Olanzapine, lorazepam, and fluoxetine Then, ECT and combination of above drugs	(i) She was discharged with lorazepam and fluoxetine. One month after discharge, revealed continued remission from catatonia but a mild return of her OCD symptoms

BPRS: Brief Psychiatric Rating Scale; HAM-D: Hamilton Depression Rating Scale; HAM-A: Hamilton Anxiety Rating Scale; YGTSS: Yale Global Tic Severity Scale; GTS: Gilles de la Tourette syndrome; YBOCS: Yates-Brown Obsessive Compulsive Symptoms; GAF: Global Assessment of Functioning.

## Data Availability

A preprint has previously been published (Soumitra Das, Sakshi Prasad, Palak Atul Fichadia, et al.: Recurrent Catatonia due to Episodic Obsessive-Compulsive Disorder: A Case Report. Authorea. August 12, 2022; doi:10.22541/au.166030588.86281061/v1).
